# Cloning and sequence analysis of hyaluronoglucosaminidase *(nagH)* gene of *Clostridium chauvoei*

**DOI:** 10.14202/vetworld.2017.1104-1107

**Published:** 2017-09-21

**Authors:** Saroj K. Dangi, Pavan Kumar Yadav, Aakanksha Tiwari, Viswas Konasagara Nagaleekar

**Affiliations:** Division of Bacteriology and Mycology, ICAR-Indian Veterinary Research Institute, Izatnagar, Bareilly - 243 122, Uttar Pradesh, India

**Keywords:** black quarter, *Clostridium chauvoei*, hyaluronoglucosaminidase

## Abstract

**Aim:**

Blackleg disease is caused by *Clostridium chauvoei* in ruminants. Although virulence factors such as *C. chauvoei* toxin A, sialidase, and flagellin are well characterized, hyaluronidases of *C. chauvoei* are not characterized. The present study was aimed at cloning and sequence analysis of hyaluronoglucosaminidase (*nagH*) gene of *C. chauvoei*.

**Materials and Methods:**

*C. chauvoei* strain ATCC 10092 was grown in ATCC 2107 media and confirmed by polymerase chain reaction (PCR) using the primers specific for 16-23S rDNA spacer region. *nagH* gene of *C. chauvoei* was amplified and cloned into pRham-SUMO vector and transformed into *Escherichia cloni* 10G cells. The construct was then transformed into *E. cloni* cells. Colony PCR was carried out to screen the colonies followed by sequencing of *nagH* gene in the construct.

**Results:**

PCR amplification yielded *nagH* gene of 1143 bp product, which was cloned in prokaryotic expression system. Colony PCR, as well as sequencing of *nagH* gene, confirmed the presence of insert. Sequence was then subjected to BLAST analysis of NCBI, which confirmed that the sequence was indeed of *nagH* gene of *C. chauvoei*. Phylogenetic analysis of the sequence showed that it is closely related to *Clostridium perfringens* and *Clostridium paraputrificum*.

**Conclusions:**

The gene for virulence factor *nagH* was cloned into a prokaryotic expression vector and confirmed by sequencing.

## Introduction

Black quarter or blackleg is a highly fatal and acute disease of mainly cattle and sheep, caused by *Clostridium chauvoei*, a Gram-positive, histotoxic, motile, and sporulating anaerobic bacteria. Cattle of 6-24 months age are commonly affected. *C. chauvoei* is one of the most pathogenic *Clostridium* species [1] causing tremendous economic losses to cattle herders [2]. There were 68 outbreaks, 547 attacks and 218 deaths in India, due to BQ from January 2016 to October 2016 alone in cattle, buffaloes, sheep, and goats [3].

Although it is presumed that *C. chauvoei* produces several toxins, molecular mechanism of pathogenicity, and the role of virulence factors in the pathogenesis of the disease is still unclear. *C. chauvoei* is presumed to produce five toxins, namely, alpha (oxygen-stable hemolysin), delta (oxygen-labile hemolysin), beta (DNase), gamma (hyaluronidase), and a neuraminidase [4,5]. Although virulence factors such as sialidase, flagellin, and *C. chauvoei* toxin A (*cctA*) are better characterized [1,5-7] other virulence factors, including hyaluronidase, remain to be characterized.

Hyaluronidases are important virulence factors that help in spread of bacteria and toxins. Bacterial hyaluronidases of pathogenic Gram-positive bacteria can break down hyaluronate. Hyaluronidases enable the spread of the pathogens from the initial site of infection to the target organs [8]. The genome of *C. chauvoei* has been shown to harbor genes for two different hyaluronidases, hyaluronoglucosaminidase (*nagH)* and β-N-acetylglucosaminidase (*nagJ)* [1]. At present, little is known about hyaluronidases of *C. chauvoei*. As a prerequisite to characterization of its role in virulence, this in the present study, we amplified, cloned, and characterized the *nagH* gene.

## Materials and Methods

### Ethical approval

Since no animal experimentation involved in this work, ethical permission is not required.

### Bacteria and vectors

The bacteria *C. chauvoei* strain ATCC 10092 (ATCC, USA); *E. cloni* 10G cells and pRham-N-His-SUMO-Kan Vector (Sigma, USA) were used in this study. The bacterial strain was cultured in ATCC 2107 media (peptone - 10.0 g, beef extract - 10.0 g, yeast extract - 3.0 g, dextrose - 5.0 g, NaCl - 5.0 g, soluble starch - 1.0 g, L-cysteine HCl - 0.5 g, sodium acetate - 3.0 g, resazurin (0.025%) - 4 ml, and DI water to make 1000 ml) at 37°C for 48 h under anaerobic conditions.

### Genomic DNA isolation

*C. chauvoei* genomic DNA was extracted using GeneJET Genomic DNA purification kit (Thermo Scientific, USA) as per manufacturer’s instructions. Briefly, *C. chauvoei* cells were centrifuged, and the obtained pellet was resuspended in 180 µl of the bacterial lysis buffer followed by incubation at 37°C. Lysis solution (200 µl) and proteinase K (20 µl) were added and the sample was incubated at 56°C. RNase A solution (20 µl) was added and incubated for 10 min. Ethanol (400 µl) was added, and lysate was added to the column, centrifuged, and flow-through was discarded. Then, wash Buffer I (500 µl) was added, centrifuged, and flow-through was discarded. Wash Buffer II (500 µl) was added to the column, centrifuged, and flow-through was discarded. Genomic DNA was eluted using elution buffer (100 µl) and stored at −20°C.

### Polymerase chain reaction (PCR) for confirmation of C. chauvoei

PCR amplification of 16S-23S rDNA was performed as described by elsewhere [9]. *C. chauvoei* was identified using IGSCS and 23UPCH primers specific for 16-23s rRNA spacer region.

### PCR amplification of hyaluronidase (nagH) gene of C. chauvoei

PCR was carried out in the volume of 25 µl standard PCR reaction mixture containing primers (For - CGCGAACAGATTGGAGGTGGTGCAGCATCAAATCCAAAT; Rev-GTGGCGGCCGCTCTATTA ACTTCTAGGAGGTCCCCCAAA) and genomic DNA template. PCR conditions used consist of initial denaturation temperature of 94°C (5 min) followed by 34 cycles of denaturation of 94°C (1 min), annealing temperature of 58°C (1 min) and extension of 72°C (1 min). Final extension was carried out at 72°C (10 min). PCR amplicons were analyzed by agarose gel electrophoresis.

### Gel extraction of PCR product

The gel containing the DNA fragment was excised using a clean scalpel and placed into a pre-weighed 1.5 ml tube and weighed. The weight of the gel slice was recorded. The gel extraction of DNA fragments was carried out using Gene JET gel extraction kit (Thermo Scientific, USA) following the manufacturer’s instructions.

### Cloning of hyaluronidase (nagH) gene

The amplified *nagH* gene was purified using MinElute gel extraction kit (Qiagen, USA) as per manufacturer’s protocol and cloned into pRham-N-His SUMO-Kan expression vector (Lucigen Corporation, USA) as per manufacturer’s protocol. Briefly, *E. cloni* 10G competent cells were thawed and transferred into a pre-cooled 15 ml centrifuge tube. 25 ng of pRham-N-His SUMO-Kan Vector DNA was added with 100 ng of the purified PCR product and mixed. The mixture of cells and DNA was incubated on ice for 30 min, heat shocked the cells at 42°C for 45 s, followed by addition of 960 µl of recovery medium and incubation at 37°C in a shaking incubator for 2 h and plated on the luria broth agar containing kanamycin (30 µg/ml). The recombinant clones were confirmed by colony PCR using the nagH specific primers and also by sequencing. Sequence was subjected to analysis using NCBI’s BLAST program. Phylogenetic tree was constructed by the neighbor-joining method with a bootstrap value of 1000 using the MEGA6 software version 6.0 [10].

## Results

**Figure-1:**
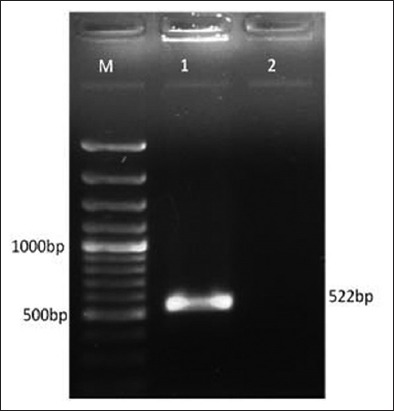
Confirmation of *C. chauvoei* by polymerase chain reaction (PCR) based on 16S-23S rDNA spacer gene: Lane M: 100 bp DNA ladder, Lane 1: PCR product (16S-23S rDNA spacer gene primers), Lane 2: Negative control.

**Figure-2:**
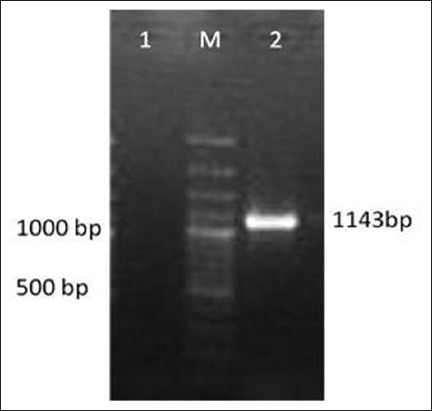
Polymerase chain reaction amplification of *nagH* gene of *Clostridium chauvoei:* Lane M: 100 bp DNA Ladder, Lane 1: Negative control, Lane 2: *nagH* gene.

*C*. *chauvoei* culture was confirmed using 16S-23S rRNA spacer region specific PCR [9], which yielded a specific amplicon of 522 bp (Figure-1). Amplification of *nagH*gene of *C. chauvoei* yielded amplification at 1143 bp (Figure-2) as expected. PCR amplified *nagH* gene was purified, and the yield of the DNA was about 80% of the PCR product.

**Figure-3:**
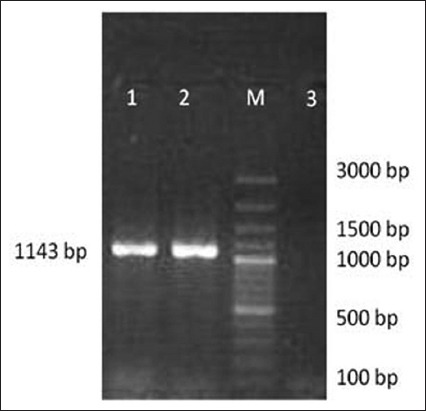
Confirmation of the recombinant clones by colony polymerase chain reaction: Lane M: 100 bp DNA Ladder, Lanes 1 and 2: *nagH* amplicon of 1143 bp size, Lane 3: Negative control.

**Figure-4:**
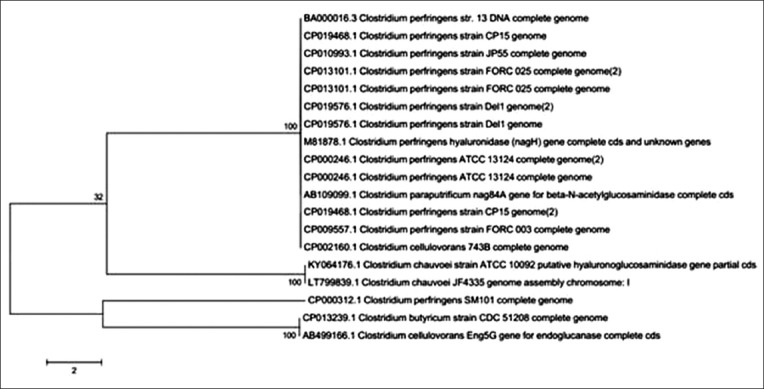
Phylogenetic analysis of *nagH* gene of *Clostridium chauvoei*.

The PCR products were cloned in pRham N-His SUMO Vector and confirmed by colony PCR using *nagH* specific primers, which yielded 1143 bp size amplicons (Figure-3). Sequence analysis confirmed that the clones were positive for *nagH* gene confirming the presence of insert in the construct. The *nagH* gene sequence was submitted to nucleotide sequence database of NCBI, which was assigned with accession number, KY064176.1. Phylogenetic analysis revealed that *nagH* gene of *C. chauvoei* ATCC 10092 was closely related to that of *C. chauvoei* JF4335 strain, as well as *Clostridium perfringens, Clostridium cellulovorans*, and *Clostridium paraputrificum* strains (Figure-4) thus indicating that the *nagH* gene of *C. chauvoei* could be having structural/functional similarity to that of other *Clostridium* species.

## Discussion

Black quarter, also known as blackleg, caused by *C. chauvoei* is a disease with high-mortality affecting cattle, sheep, and other animals. *C. chauvoei* secretes exotoxins which are protein in nature and responsible for the pathogenesis of black quarter such as α toxin (oxygen stable hemolysin), β toxin (DNase), γ toxin (hyaluronidase), δ toxin (oxygen labile hemolysin), neuraminidase (sialidase), and *cctA* [11]. Few genes of *C. chauvoei* such as cctA [6], sialidase NanA [5], NAD-dependent beta hydroxybutyryl CoA dehydrogenase [12] have been cloned, sequenced, and expressed. However, only little is known about other virulence factors, molecular mechanisms of pathogenicity and the role of various toxins in the pathogenesis of the disease. *C. chauvoei* produce an enzyme hyaluronidase, which degrades the connective tissue substance called hyaluronate in the host. As revealed in another study, the genome of *C. chauvoei* harbors two different genes for hyaluronidase, namely, *nagH* and *nagJ* [1]. Hyaluronidase is shown to be involved in degrading the intracellular matrix and tissue connections so that *C. chauvoei* and its toxins can spread in the infected host. Hence, we selected this gene for characterization in *C. chauvoei*.

Sequence analysis of the hyaluronidase genes indicated that *nagH* gene of *C. chauvoei* ATCC 10092 was closely related to that of *C. chauvoei* JF4335 strain, as well as *C. perfringens* and *C. paraputrificum* strains. *C. perfringens*, genome sequence, has five hyaluronidase genes [13] as compared to two hyaluronidase genes in case of *C. chauvoei*. All of *nagH* genes reported so far, have revealed N-terminal signal sequences, indicating the secretory nature of these enzymes [14]. *C. paraputrificum* has been shown to harbor *nag84A* gene, which encodes for hyaluronidase, though it did not have hyaluronidase activity [15].

## Conclusion

In this study, *nagH* gene was amplified and cloned into prokaryotic expression vector, and recombinant clones were confirmed by colony PCR followed by sequencing. However, more research is needed to express the *nagH* gene and to characterize its role in virulence of *C. chauvoei*.

## Authors’ Contributions

SKD carried out the laboratory work, and VKN designed the work. SKD and VKN drafted and revised the manuscript. PKY and AT assisted in the experiments.
